# Non-Classical ProIL-1beta Activation during Mammary Gland Infection Is Pathogen-Dependent but Caspase-1 Independent

**DOI:** 10.1371/journal.pone.0105680

**Published:** 2014-08-27

**Authors:** Koen Breyne, Steven K. Cool, Dieter Demon, Kristel Demeyere, Tom Vandenberghe, Peter Vandenabeele, Harald Carlsen, Wim Van Den Broeck, Niek N. Sanders, Evelyne Meyer

**Affiliations:** 1 Department of Pharmacology, Toxicology and Biochemistry, Faculty of Veterinary Medicine, Ghent University, Merelbeke, Belgium; 2 Department of Nutrition, Genetics and Ethology, Faculty of Veterinary Medicine, Ghent University, Merelbeke, Belgium; 3 Department of Medical Protein Research, Vlaams Instituut voor Biotechnologie (VIB), Ghent University, Ghent, Belgium; 4 Department for Molecular Biomedical Research, Vlaams Instituut voor Biotechnologie (VIB), Ghent University, Zwijnaarde, Belgium; 5 Department of Nutrition, Institute of Basic Medical Sciences, University of Oslo, Oslo, Norway; 6 Department of Morphology, Faculty of Veterinary Medicine, Ghent University, Merelbeke, Belgium; University of São Paulo, Brazil

## Abstract

Infection of the mammary gland with live bacteria elicits a pathogen-specific host inflammatory response. To study these host-pathogen interactions wild type mice, NF-kappaB reporter mice as well as caspase-1 and IL-1beta knockout mice were intramammarily challenged with *Escherichia coli* (*E. coli*) and *Staphylococcus aureus* (*S. aureus*). The murine mastitis model allowed to compare the kinetics of the induced cytokine protein profiles and their underlying pathways. *In vivo* and *ex vivo* imaging showed that *E. coli* rapidly induced NF-kappaB inflammatory signaling concomitant with high mammary levels of TNF-alpha, IL-1 alpha and MCP-1 as determined by multiplex analysis. In contrast, an equal number of *S. aureus* bacteria induced a low NF-kappaB activity concomitant with high mammary levels of the classical IL-1beta fragment. These quantitative and qualitative differences in local inflammatory mediators resulted in an earlier neutrophil influx and in a more extensive alveolar damage post-infection with *E. coli* compared to *S. aureus*. Western blot analysis revealed that the inactive proIL-1beta precursor was processed into pathogen-specific IL-1beta fragmentation patterns as confirmed with IL-1beta knockout animals. Additionally, caspase-1 knockout animals allowed to investigate whether IL-1beta maturation depended on the conventional inflammasome pathway. The lack of caspase-1 did not prevent extensive proIL-1beta fragmentation by either of *S. aureus* or *E. coli*. These non-classical IL-1beta patterns were likely caused by different proteases and suggest a sentinel function of IL-1beta during mammary gland infection. Thus, a key signaling nodule can be defined in the differential host innate immune defense upon *E. coli* versus *S. aureus* mammary gland infection, which is independent of caspase-1.

## Introduction

Infectious mastitis is defined as the inflammatory response initiated when microorganisms enter the mammary gland challenging the host defense [1]. This common disease has either clinical or asymptomatic (subclinical) characteristics and is generally perceived as a significant burden for the well-being of mammals and especially dairy animals [2]. Two main bacterial species that cause bovine mastitis are *Escherichia coli* (*E. coli*) and *Staphylococcus aureus* (*S. aureus*) [3]. Although both these pathogens grow in the mammary gland evoking a host immune response, they activate specific inflammatory signaling pathways which result in discriminatory stress profiles [4], [5], [6]. This distinctive pathobiology can be explained by the microbe-associated molecular patterns (MAMPs) dictating the expression and subsequent release of specific pro-inflammatory cytokines [7], [8]. In the initial phase, these mediators orchestrate the diapedesis of predominantly neutrophils into the mammary alveoli activating phagocytic innate immune cells to eliminate pathogens or at least prevent their spreading [9], [10], [11]. Most of the observations seen in cows have been further elaborated at the molecular level through *in vivo* studies in mouse mastitis models [12], [13].

Our group previously reported that *in vitro* exposure of bovine neutrophils to live *E. coli* rapidly activates a complex series of molecular pathways involving cell death, the cleaving of the protease procaspase-1 and the transcription factor nuclear factor-kappaB (NF-kappaB). This activity occurs concomitant with the secretion of the pro-inflammatory cytokine IL-1beta [14], [15]. Two studies from our group using intramammary infections (IMI) in mice confirmed the relevance of these key parameters *in vivo* [16], [17]. However, elucidation of the link between these innate mammary host defense factors and their relevance for other mastitis pathogens than coliforms is just starting to emerge. It was already demonstrated for the bovine species that both *E. coli* and *S. aureus* bind mammary epithelial Toll-like receptor (TLR)4 and TLR2, but that both these pathogens differently modulate NF-kappaB [18]. Indeed, *E. coli* increases mammary epithelial NF-kappaB activity through myD88-dependent signaling followed by enhanced mRNA cytokine expressions [18]. In contrast, mammary *S. aureus* infections are characterized by reduced local NF-kappaB levels. These observations are linked to the internalization of these bacteria in bovine epithelial cells [19]. To date, NF-kappaB transcriptional activity is accepted to induce proIL-1beta transcription and the subsequent release of pro-inflammatory cytokines either locally and/or systemically. Nevertheless, reported mastitis data are still partly contradictory as they on the one hand state that active IL-1beta predominates upon infection with *S. aureus* compared to *E. coli*, while adverse data have also been published [20], [21]. Additionally, it was recently shown that total IL-1 signaling is of critical importance for the proper influx of neutrophils into the alveolar lumen following an infection with *E. coli* [9]. Overall, little is known about the source of proIL-1beta or about its maturation process during mastitis. In a variety of other infectious pathologies, the production of this inactive pro-form is mediated by NF-kappaB, while its biological activity is regulated by a cytoplasmic multi-protein complex named the inflammasome. The inflammasome binds procaspase-1 and enables its activation; in turn the latter protease activity mediates the maturation of proIL-1beta [22].

The current *in vivo* study aims to further elucidate the main mechanistical differences or similarities between mammary *S. aureus* or *E. coli* infections as a basis for novel intervention strategies. Key findings highlight the non-classical maturation of pro-IL1beta independently of caspase-1 during mammary inflammation. The resulting cleavage patterns were pathogen-specific and occurred concomitant with important steps in the innate immune response of the mammary gland such as the NF-kappaB activation, the expression of specific cytokine profiles, the influx of neutrophils and changes in the integrity of the epithelial layer.

## Materials and Methods

### Mice

Female albino NF-kappaB *luc* mice were kindly provided by Harald Carlsen (University of Oslo, Department of Nutrition, Institute of Basic Medical Sciences) [23]. Genetically these mice are heterozygous for the NF-kappaB *luc* transgene in a Tyr^−^/^−^ C57BL/6 background and thus easier to visualize for *in vivo* imaging purposes than Tyr^+^/^+^ animals. Caspase-1^−^/^−^ caspase-11^−^/^−^ double knockout and IL-1beta^−^/^−^ single knockout (KO) mice also have a C57BL/6 background and were kindly provided by Tom Vandenberghe. (Ghent University, Molecular Signaling and Cell Death Unit, Department for Molecular Biomedical Research, VIB). The latter were compared with wild type (wT) C57BL/6 mice provided by Harlan. All mice were conventionally housed with water and food supplied ad libitum, and maintained with a 12 hours (h) light/dark cycle. Sentinels were routinely screened to verify the pathogen-free conditions. This study was carried out in strict accordance with the recommendations in the Guide for the Care and Use of Laboratory Animals of the National Institutes of Health. The protocol was approved by the Committee on the Ethics of Animal Experiments of the Ghent University (Permit Number: EC2011/042 and EC2010/148). All efforts were made to minimize suffering. Surgery was performed under isoflurane anesthesia combined with a long-acting analgesic buprenorphine, while a ketamine and xylazine containing cocktail was administered prior to euthanasia.

### Intramammary infection model

Eight-week-old mice (wT or transgenic mice) were allowed to mate with a ten-week-old male. Following parturition, the pups were weaned only after ±10 days to enhance mammary gland development. One hour after weaning, mice were intramammarily inoculated using a 32-gauge blunt needle (fourth gland pair) with 3.20 × 10^3^±374.6 CFU *S. aureus* Newbould 305 or *E. coli* P4:032, or PBS under inhalational anesthesia. These bacteria are relevant cow mastitis isolates inducing a specific inflammatory response in our established mouse mastitis model [16], [24].

During the Intramammary injection (IMI) experiment, core body temperature of the mice was measured with a rectal thermistor and compared to the body temperature pre-IMI. Blood was harvested through the tail vain or cardiac puncture, incubated for 1 h at 37°C and centrifuged (12250 g) for another 1 h. Following sacrifice (cervical dislocation) the inoculated glands (and untreated livers) were isolated, homogenized and plated in serial logarithmic dilutions on Tryptic soy agar plates to determine CFU/g gland. Supernatant/serum samples were stored individually at −80°C until use.

### *In vivo* and *ex vivo* bioimaging

Imaging of luciferase activity in intramammary injected transgenic mice was performed with the IVIS lumina II (Caliper) [25]. To visualize the NF-kappaB signal a suspension of D-luciferin (2 mg/100µl) dissolved in PBS was injected. NF-kappaB *luc* mice were pre-anesthetized by isoflurane, injected right and left of the abdomen with luciferin, and imaged about 10 minutes following injection. Briefly, bioluminescence is visualized when NF- kappaB gets activated, translocates to the nucleus where it binds the kappaB sites and transcribes the luciferase gene. As a result the injected luciferin is oxidized by the newly produced luciferase together with ATP, which yields a bioluminescent light signal. The transgenic mice were measured before (at -2 h) and kinetically after IMI (at 2 h, 4 h, 8 h, 10 h, 12 h and 24 h). At 12 h (n = 11) and 24 h (n = 32) mice were sacrificed, organs harvested and separately visualized through imaging. The intensity of the bioluminescent signal is linked with the level of NF-kappaB activity and normalized through the division of the total flux data by the selected area as provided by the living image software 3.2 (Caliper).

### Cytokine analysis

Hundred microliters of mammary gland homogenate were mixed with 200 µl lysis buffer supplemented with protease inhibitors (200 mM NaCl, 10 mM Tris-HCl pH 7.4, 5 mM EDTA, 1% Nonidet P-40, 10% glycerol, 1 mM oxidized L-glutathion, 100 µM PMSF, 2.1 µM leupeptin and 0.15 µM aprotinin) to extract cellular proteins. The suspensions rested overnight at −20°C, were centrifuged (12.250 g) for 1 h and finally the supernatant was centrifuged for another 30 minutes (min) to precipitating the pellet. The protein concentration in the supernatant was spectrophotometrically (Genesys 10S) determined with Bio-Rad Protein Assay (Biorad). Cytokine quantification in the lysates and serum was performed with specific Cytometric Bead Array kits (CBA, Becton Dickinson) for mouse IL-6, MCP-1, TNF-alpha, and IL-1alpha and specific Aimplex multiplex assay kits (YSL Bioprocess Development Co.) with minor modifications for mouse KC and MIP-2 on a FACSArray instrument (Becton Dickinson). Fifty μg lysate or ¼ diluted serum were applied to each well.

### Immunohistochemistry

Two mammary glands per condition (isolated from the NF-kappaB *luc* mice) were fixed in buffered 3.5% formaldehyde for 24 h at room temperature and embedded in paraffin wax. Sections were deparaffinized, hydrated, and antigens retrieved in a citrate buffer through microwaves and cooker pressure for p50 and p65, respectively. To mask endogenous peroxidase activity, specimens were pre-treated with 3% hydrogen peroxide in methanol for 5 min at room temperature. Non-specific binding sites were blocked by 30% goat serum and 1% BSA for 30 min at 25°C. The tissues were incubated overnight at 4°C with p65 (rabbit anti mouse, sc-372, Santa Cruz) and p50/p105 (rabbit anti-mouse, ab7971, Abcam). The next day, samples were incubated with a biotinylated secondary antibody (goat anti-rabbit) that binds streptavidin conjugated to horseradish peroxidase (HRP). A 3,3′-Diaminobenzidine chromogen generates a brownish peroxidase-based signal visualized through the HRP reaction together with a hematoxylin counterstain. All rinsing steps were performed with Tris-buffered saline (TBS) supplied with Triton-X 100.

### Western analysis

Mammary gland lysates (prepared as described sub cytokine analysis) were loaded in equal volumes (18 µg protein/20 µl buffer), proteins were separated on a 12% polyacrylamide Amersham ECL Gel (GE Healthcare) and the proteins transferred to a 0.45 µm nitrocellulose transfer membrane (Biorad). A donkey anti-goat IgG (Bioconnect) was used to detect IL-1beta (R&D) and donkey anti-rabbit IgG (GE Healthcare) was used to detect caspase-1 (kindly provided by Wim Declercq).

### Statistical analysis

P-values of normal distributed data were calculated by ANOVA testing. Depending on the Levene's test, mean values were compared by Tukey post hoc comparisons. If necessary, normalization occurred through log10 transformation and if normalization was impossible, median values were compared with non-parametic statistics and Mann-withney U post hoc-testing.

## Results

### The intramammary bacterial growth post-intramammary infection with *S. aureus* and *E. coli*

**Figure 1:**
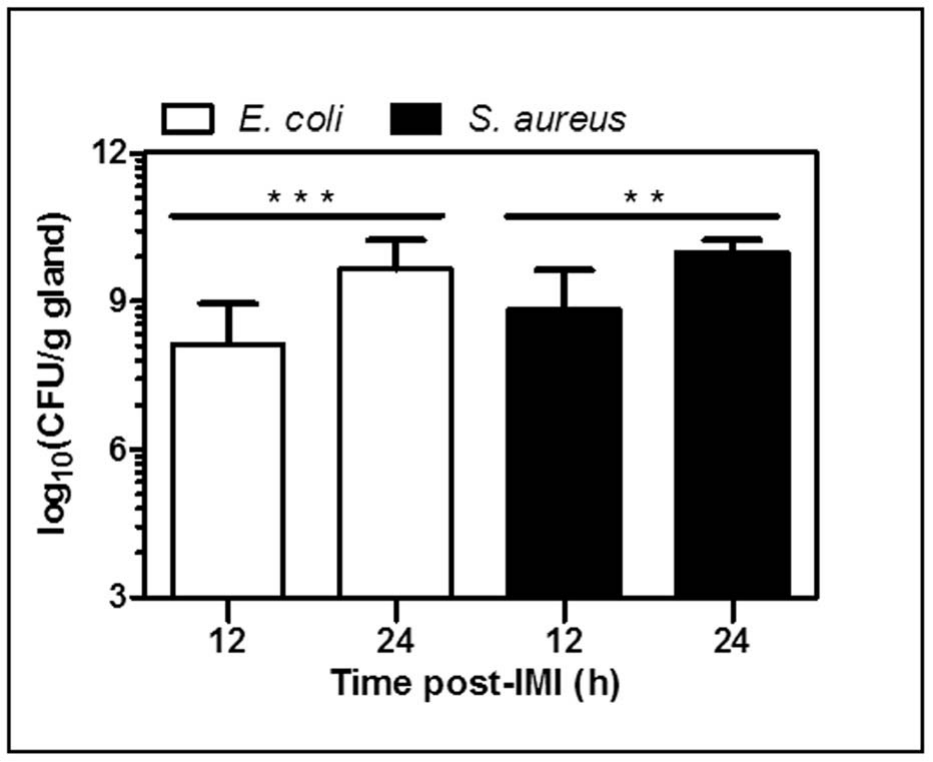
Changes in mammary bacterial counts post-IMI with *E. coli* or *S. aureus*. At 12 h and 24 h post-IMI, mammary tissue was harvested and evaluated for bacterial growth. (P<0.01 **, P<0.001***)

Sham-inoculated (PBS) mice (n = 9; data not shown) were compared to mice intramammarily inoculated with (3.203±0.38) × 10^3^ CFU of either *E. coli* (n = 12) or *S. aureus* (n = 12). Both at 12 h and 24 h post-IMI, mammary glands were isolated and their CFU count was determined (Fig. 1). There was no statistically significant median difference (MD) between both treatment groups at the two time points of sacrifice. Nevertheless, significant intramammary growth of each of both pathogens was detected between these time points (*E. coli*, P<0.001; *S. aureus*, P<0.01).

### Mammary cytokine profiles post-intramammary infection with *E. coli* compared to *S. aureus*

Differential cytokine patterns were observed in the mammary gland post-IMI with *E. coli* versus *S. aureus* at 12 h or 24 h (n = 10 for all groups).

**Figure 2:**
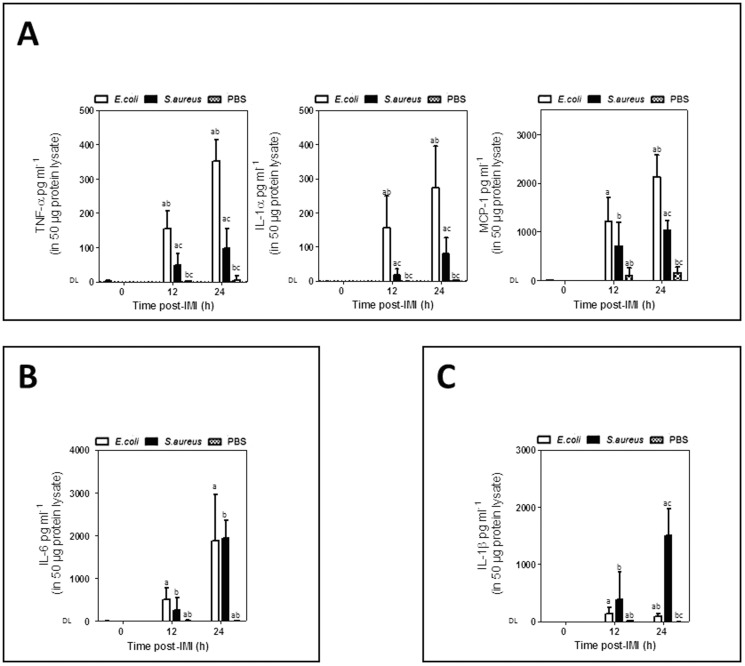
Differential mammary innate immune response post-IMI with *E. coli* versus *S. aureus*. (A) Local TNF-alpha, IL-1alpha, and MCP-1 concentrations in lactating mammary glands compared to mammary glands post-IMI with *E. coli, S. aureus* or sham-inoculated (PBS). (B) Local IL-6 concentrations in lactating mammary glands compared to mammary glands post-IMI with *E. coli* or *S. aureus* or sham-inoculated (PBS) mice. (C) Local concentrations of active IL-1beta in lactating mammary glands compared to mammary glands post-IMI with *E. coli, S. aureus* or sham-inoculated (PBS) mice. Letters of homogeneous subsets were marked if the difference between treatments were statistically significant (P<0.001) (DL = detection limit).

At the local level, *E. coli* dominated the innate immune response on both time points by a strong and continuing increase of TNF-alpha (at 12 h, P<0.001; at 24 h, P<0.001), IL-1alpha (at 12 h, P<0.001; at 24 h, P = 0.001) and MCP-1 albeit only at the later time point (at 12 h, P = 0.051; at 24 h, P = 0.001) compared to *S. aureus* (Fig. 2A). Concentrations of these cytokines in all untreated lactating glands (at 0 h, n = 6) and sham-inoculated glands (PBS, n = 8 at 12 h and at 24 h) at 12 h and 24 h remained below the detection limit (DL). Absolute MCP-1 levels were up to 8-fold higher than those of TNF-alpha and IL-1alpha. The increase of TNF-alpha and IL-1alpha was similar when comparing *E. coli* to *S. aureus* (R^2^ = 0.81; P<0.001) i.e. about 3-fold higher for *E. coli* compared to *S. aureus*, while this relative difference between both pathogens was about 2-fold for MCP- 1 (Fig. 2A).

An IMI with both pathogens induced a strong and continuing local increase in IL-6 (Fig. 2B).

In marked contrast to TNF-alpha, IL-1alpha and MCP-1, this response did not significantly differ between the *E. coli* and *S. aureus* but did significantly differ for each pathogen compared to the sham-inoculated (PBS) glands (at 12 h, P<0.001; at 24 h, P<0.001) and lactating glands (at 0 h, P<0.001).

Post- IMI with *S. aureus*, a strong response albeit only at a later time point was observed for IL-1beta. At 12 h post-IMI local levels were low and did not differ significantly between both pathogens, while at 24 h post-IMI *S. aureus* strongly induced high local levels of this cytokine compared to *E. coli* (P<0.001) (Fig. 2C). Concentrations of IL-1beta in the untreated lactating glands (at 0 h) and sham-inoculated glands (at 12 h and 24 h) remained below the DL (detection limit). Pathogen-specific profiles in serum or liver samples were also detected and are discussed in the supporting information (see Fig S1A and S1B).

### Maturation of proIL-1beta and procaspase-1 post-intramammary infection with *E. coli* compared to *S. aureus*

The observed quantitative differences in cytokines do not necessarily imply biological activity of these proteins as for several cytokines this is the result of post-translational modification of their proforms. More specifically, activation of proIL-1beta is achieved by cytoplasmic multi-protein complexes named inflammasomes. The best known inflammasome enables procaspase-1 (p45) maturation upon vita-MAMP recognition. This active caspase-1 then mediates the maturation of active IL-1beta as characterized by its classical p17 fragment [22].

**Figure 3:**
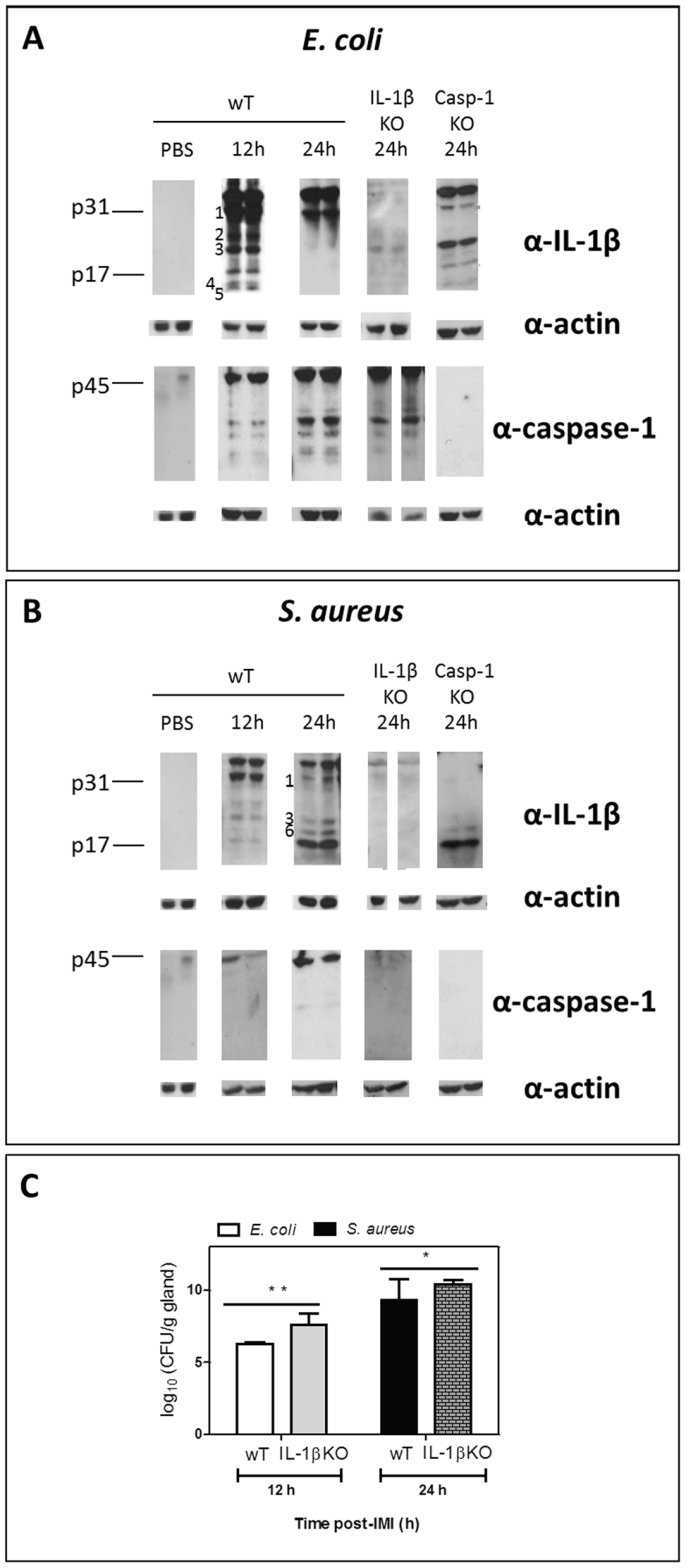
Differential mammary IL-1beta fragmentation post-IMI with *E. coli* versus *S. aureus* and effect on bacterial growth. (A) Cleavage of IL-1beta in the mammary gland post-IMI with *E. coli* shows a fast, transient IL-1beta maturation with six IL-1beta fragments at 12h i.e. ±30 kDa, 25 kDa, ±20 kDa, ±17 kDa, ±15 kDa and ±10 kDa (fragments 1, 2, 3, p17, 4 and 5, respectively). At 24 h (only fragment 1 and p31 proform) is seen. In IL-1beta KO and in sham-inoculated (PBS) mice no fragments or p31 proform are detected. Despite clear procaspase-1 maturation, the early complex IL-1beta pattern is not the result of caspase-1 cleavage as the latter only occurs extensively at 24 h and as cleavage of pro-IL-1beta still occurs in caspase-1 KO glands (right Western blot images). No caspase-1 maturation was detected in caspase-1 KO or in sham-inoculated mammary glands. (B) Cleavage of IL-1beta in the mammary gland post-IMI with *S. aureus* shows a slower IL-1beta maturation with four IL-1beta fragments at 24 h i.e. ±30 kDa, ±20 kDa, ±18 kDa and ±17 kDa (fragments 1, 3, 6 and p17, respectively). At 12 h (only fragment 1 i.e the preform p31, 2, 3,and p17). The late IL-1beta maturation is not the result of caspase-1 cleavage as IL-1beta cleavage still occurs in caspase-1 KO glands (strong p17 fragment albeit in the absence of the p31 proform). Both procaspase-1 and its cleavage were low, respectively absent in IL-1beta KO glands (right Western blot images). No IL-1beta or caspase-1 was detected in sham-inoculated mammary glands of wT mice. (C) ProIL-1beta fragmentation affects bacterial growth as in IL-1beta KO mammary glands on time points of interest (12 h for *E. coli*, 24 h for *S. aureus*) CFU counts are significantly higher for both pathogens than in wT mammary glands, especially for *E. coli* (P<0.01 *, P<0.001**). DL = detection limit.

Based on multiplex results the local active IL-1beta concentration at 24 h was higher post-IMI with *S. aureus* than with *E. coli* (Fig. 2C). This quantitative analysis measures the number of active p17 fragment. The absence of mammary IL-1beta in IL-1beta KO animals was confirmed at both time points through multiplex analysis, no significant differences in the local levels of other cytokines could be detected either (data not shown). However, using Western blot analysis as a complementary immunoassay, the complete fragmentation pattern of mammary proIL-1beta was evaluated post-IMI with *E. coli* compared to *S. aureus* (Fig. 3A and B).

Multiplex analysis showed that an IMI in wT mice with *E. coli* induced the fast but transient increase of active IL-1beta (p17) compared to sham-inoculated (PBS) mammary glands (at 12 h, P<0.001; at 24 h, P<0.001) (Fig. 2C). Western blotting of these samples revealed that this increase of p17 the result of a fast proIL-1beta (p31 band, fragment 1) maturation into multiple fragments which were absent post-IMI with PBS. Indeed, at 12 h post-IMI a complex pattern of at least six IL-1beta fragments were detected concomitant with procaspase fragmentation i.e. at ±30 kDa, ±25 kDa, ±20 kDa, ±17 kDa, ±15 kDa and ±10 kDa (fragments 1, 2, 3, p17, 4 and 5, respectively; Fig. 3A). In marked contrast, only one band (i.e. fragment 1, p31) of the proIL-1beta maturation remained at 24 h post-IMI with *E. coli* although procaspase-1 cleavage was induced at that later time point (Fig. 3A).

As expected, IL-1beta fragments were largely absent in similarly infected IL-1beta KO mammary glands while procaspase-1 fragmentation was still observed (Fig. 3A). Interestingly, extensive proIL-1beta (p31) maturation was also seen in caspase-1 KO mammary glands at 12 h albeit with a lower number of bands (i.e. no fragment 2; Fig 3A).

Multiplex analysis further showed that IMI with *S. aureus* also induced active IL-1beta (p17) compared to sham-inoculated (PBS) mammary glands (at 12 h, P<0.01; at 24 h, P<0.001) (Fig. 2C). This raise of p17 occurred predominantly at 24 h and was also higher than post-IMI with *E. coli* (P<0.001). Western blotting of wT glands confirmed this late increase of p17 (Fig. 3B). While the fragmentation pattern at 12 h post-IMI with *S. aureus* was similar to that of *E. coli* (except for the fragments 4 and 5) the intensity of the bands was lower. In marked contrast, a different cleavage pattern was seen at 24 h post-IMI with *S. aureus* compared to that at 12 h post-IMI with *E. coli* (there was an additional band, fragment 6), this maturation was now totally caspase-1 independent as very limited fragmentation of procaspase-1 was observed. Compared to post-IMI with *E. coli*, the IL-1beta pattern contained a lower number of fragments post-IMI with *S. aureus* i.e. at ±30 kDa, 20 kDa, ±18 kDa and 17 kDa (corresponding to fragments 1, 3, 6 and p17, respectively; Fig. 3B). As for *E. coli*, these IL-1beta fragments were all absent in similarly affected IL-1beta KO mammary glands. Again in marked contrast to IMI with *E. coli*, no procaspase-1 was observed at 24 h in IL-1beta KO mammary glands while an extensive IL-1beta maturation (p17 fragment) was seen in caspase-1 KO mammary glands post-IMI with *S. aureus.*

When comparing bacterial growth in IL-1beta KO mammary glands to their wT counterparts at the selected relevant time points for each of both pathogens, a significantly higher number of CFU was seen for *E. coli* and to a lower extent also for *S. aureus* (P<0.01, n = 4 and P<0.05, n = 6 Fig. 3C).

### The mammary NF-kappaB activity post-intramammary infection with *E. coli* compared to *S. aureus*

**Figure 4:**
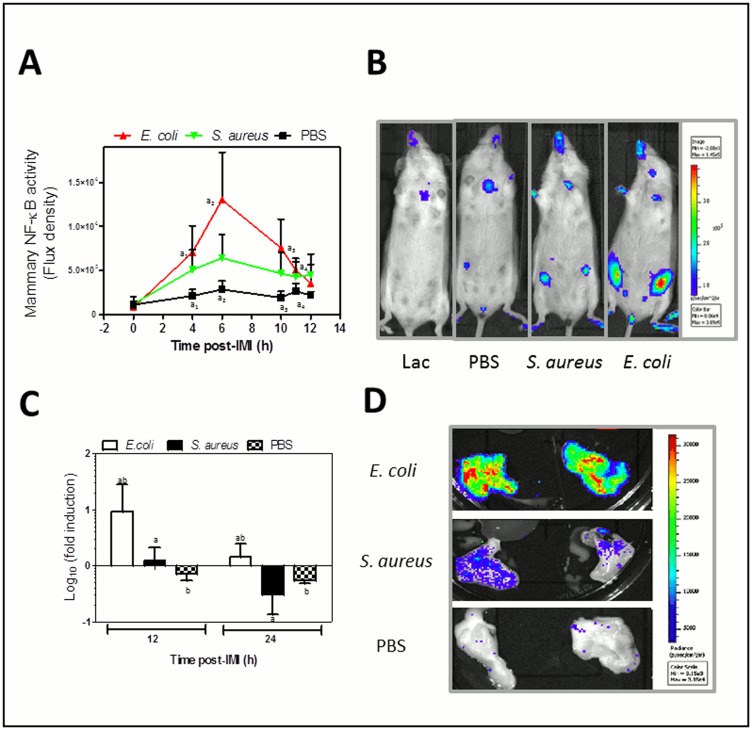
*In vivo* mammary NF-kappaB activity post-IMI with *E. coli* compared to *S. aureus*. (A) *In vivo* imaging of local NF-kappaB activity in mammary glands (ventral view) showed a transient and fast activation for both pathogens compared to sham-inoculated (PBS) glands and also about a 3-fold higher activation post-MI with *E. coli* compared to *S. aureus*. Data represent the flux density (total flux radiance per selected body area (in p*(s*m^2^))) as a quantitative measure of the bioluminescent signal correlating with the NF-kappaB activity. (B) A representative photograph of lactating mice (at 0 h, most left) compared to mice at 6 h post-IMI with sham (PBS, 2nd from left), at 6 h post-IMI with *S. aureus* (3rd from left), or at 6 h post-IMI with *E. coli* (most right). The intensity of luminescence was scaled based on the radiance (in p*(s*m^2^)). (C) Mammary glands of NF-kappaB reporter mice were excised at 12 h and 24 h post-IMI and their *ex vivo* luminescence was measured. The graph represents the ratio between the total flux radiance per area (in p*(s*m^2^)) at 4 h of the inoculated gland and the value from the non-injected 3th gland in the same mouse. Although the maximal activation occurred earlier, the local NF-kappaB activity was still significantly higher in *E. coli* compared to *S. aureus* mammary glands at both these time points of relevance for cytokine transcription. (D) Representative photographical view of excised mammary glands post-IMI with *E. coli* (top), *S. aureus* (middle) or after sham-inoculation (PBS, bottom). Letters of homogeneous subsets were marked if the difference between treatments were statistically significant (P<0.05).

Our group at first described the fast and transient induction of mammary NF-kappaB transcription post-IMI with *E. coli* [16]. Monitoring the NF-kappaB activity with *in vivo* imaging in reporter mice, a gradual and significant increase was again observed at 4-11h post-IMI with *E. coli* and at first also with *S. aureus* albeit non-significant (Fig. 4A). The maximal NF-kappaB activity was about a 3-fold higher post-IMI with *E. coli* compared to *S. aureus*. This correlated with the local TNF-alpha and IL-1alpha levels that were stronger upregulated post-IMI with *E. coli*, compared to *S. aureus*. No significant increase was seen in the sham-inoculated (PBS, n = 6) glands compared to pre-IMI. The increase in mammary NF-kappaB activity at 4h, at 6 h, at 8 h, at 10 h and at 11 h post-IMI was stronger for *E. coli* (P<0.01, n = 8) compared to sham-inoculated glands. Upon *in vivo* imaging at 12 h post-IMI this difference no longer remained, while *ex vivo* imaging still displayed a significant difference compared to sham inoculated glands at 12 h post-IMI (P<0.01) and 24 h post-IMI (P<0.01; Fig. 4C and D). Although not significant *in vivo*; there was a difference between *E. coli* infected and *S. aureus* infected glands (n = 8) through *ex vivo* imaging at 12 h post-IMI (P<0.05) and at 24 h post-IMI (P<0.001).

### Histology confirms the differential local NF-kappaB activation, demonstrates a delayed immune cell influx and less damage of the mammary epithelium post-IMI with *S. aureus* compared to *E. coli*

NF-kappaB-mediated transcription is only possible through translocation of its subunits to the nucleus. Immunohistochemistry was performed at 12 h and 24 h post-IMI with both pathogens on mammary gland sections.

**Figure 5:**
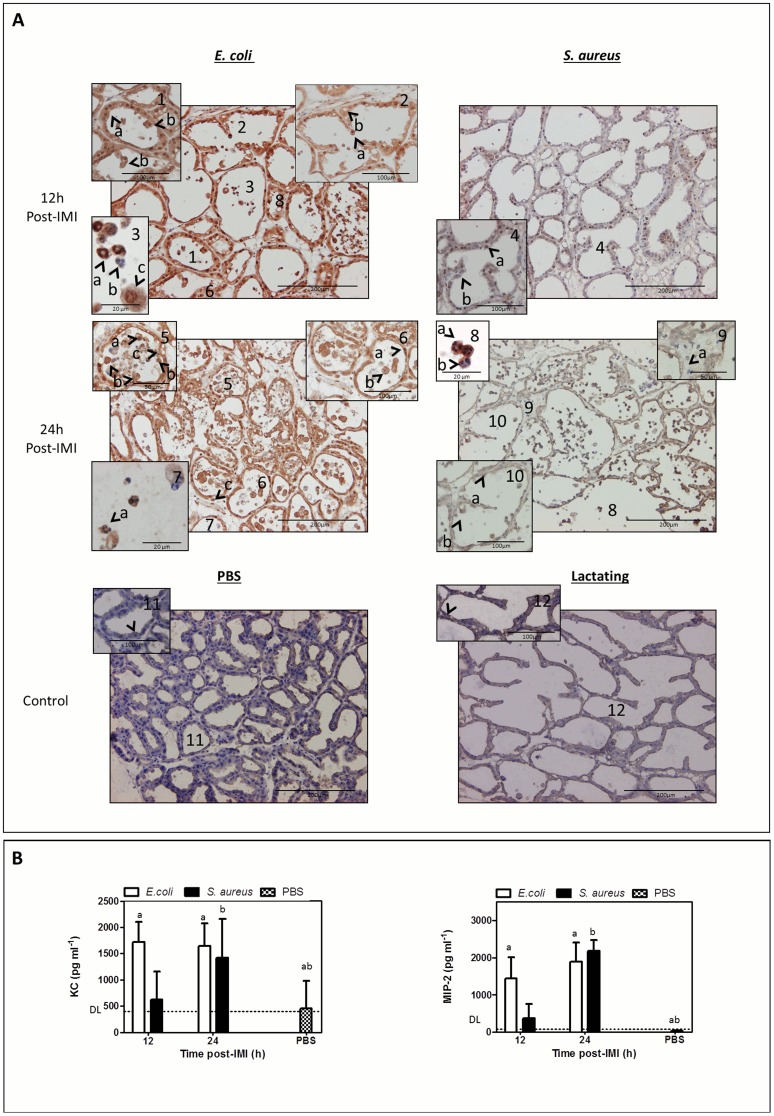
Differential nuclear translocation of mammary NF-kappaB p65, immune cell influx and damage at the mammary epithelium post-IMI with *S. aureus* compared to *E. coli*. (A) At 12 h post-IMI, a nuclear translocation of NF-kappaB subunit p65 was observed in the epithelia of both *E. coli* (-1a-) and *S. aureus-*infected glands (-4a-). At this early time point, a strong cytoplasmatic NF-kappaB p65 signal was only seen post-IMI with *E. coli*. At 24 h post-IMI, NF-kappaB p65 was mainly detected in the cytoplasm in the mammary epithelium of both *E. coli-*(-6-) and *S. aureus-*infected glands (-10-). while at this time both sham-inoculated (PBS, -11-) and lactating (at 0 h,-12-) glands displayed very low basal levels of latent expressed cytoplasmic NF-kappaB p65. At 12 h post-IMI with *E. coli* the polymorphonuclear cells in the alveolar lumen could morphologically be identified as neutrophils (-3a- and -3b-), while these immune cells were still absent at that time post-IMI with *S. aureus* (-4-) or in sham-inoculated (PBS, -11-) and lactating (at 0 h,-12-) glands. In contrast, the influx of neutrophils could only be detected at 24 h post-IMI with *S. aureus* (-8-). A clear nuclear translocation of NF-kappaB p65 could be detected at 12 h post-IMI with *E. coli* and 24 h post-IMI with *S. aureus* (-3a- and -8a-) in these immune cells. At 24 h post-IMI with *E. coli*, epithelial damage at mammary alveoli was characterized by budding of mammary epithelia (-5b-) and resulted in shedded epithelial cells (-5a-) and empty spots in the luminal layer (-5c-), while at 24 h post-IMI with *S. aureus* only some cells started to protrude from the epithelial layer (-10b-). (B) IL-8 like chemokines (i.e. KC and MIP-2) confirmed this difference in kinetics of the neutrophil influx between both pathogens. Both chemokines were significantly induced in *E. coli* infected glands compared to sham-inoculated glands (PBS) already at 12 h post-IMI (a), while for *S. aureus* this strong induction was only seen at 24 h post-IMI (b). Letters of homogeneous subsets were marked if the difference between treatments were statistically significant (P<0.05)

The NF-kappaB p65 subunit was detected in the nucleus of mammary epithelial cells and immune cells post-IMI with *E. coli* (Fig. 5A: -1a- and -3b-) as well as post-IMI with *S. aureus* (Fig. 5A: -4a- and -8a-) but was absent in both sham-inoculated (Fig. 5A -11-) and lactating mammary glands (Fig. 5A -12-). As suggested by the NF-kappaB activity imaging data, the nuclear NF-kappaB subunit p65 in the mammary epithelia was mainly visualized at 12 h and less pronounced at 24 h post-IMI with *E. coli* (Fig. 5A; -1a- compared to -6a-) and post-IMI with *S. aureus* (Fig. 5A; -4a- compared to -10a-). In marked contrast to post-IMI with *E. coli*, the p65 subunit couldn’t be detected in all nuclei of the epithelial cells at 12 h post-IMI with *S. aureus* (Fig. 5A; -4a- compared to -4b-) again confirming the higher NF-kappa activity post-IMI with *E. coli* compared to post-IMI with *S. aureus*.

Small cells with a characteristic multilobular polymorphonuclear morphology of neutrophils were detected post-IMI with *E. coli* (Fig. 5A: -3a- and -7a-) and *S. aureus* (Fig. 5A -8a-) but not in sham-inoculated (Fig. 5A -11-) or in lactating mammary glands (Fig. 5A -12-). At 12 h post-IMI with *E. coli* the alveolar lumen contained a mixture of neutrophils either with (Fig. 5A -3a-) or without (Fig. 5A -3b-) nuclear p65 the NF-kappaB subunit. However, at 24 h post-IMI, no p65 subunit was detected in these neutrophils (Fig. 5A -7a-). In contrast, for *S. aureus*, neutrophils could only be detected at 24 h post-IMI and with (Fig. 5A -8a-) or without (Fig. 5A -8b-) nuclear p65 subunit. Remarkably, post-IMI with *S. aureus* also cells without nuclei (likely red blood cells) were observed outside the blood vessels, which was not the case post-IMI *E. coli* (Fig. 5A -c-).

This observed delay in neutrophil influx post-IMI with *S. aureus* was confirmed by quantitative IL-8-like (i.e. KC and MIP-2) multiplex analysis (Fig. 5B). At 12 h post-IMI, there was already a significant difference for *E. coli* compared to sham-inoculated glands (PBS) for both these chemokines which persisted at 24 h post-IMI (P<0.01 and P<0.001, respectively). In marked contrast, post-IMI with *S. aureus* this IL-8 like chemoattractant signaling was delayed until 24 h for both chemokines compared to sham-inoculated glands (P<0.05 and P<0.001, respectively).

Additionally, as a result of the strong *E. coli-*inflicted immune response, the mammary epithelium was damaged compared to sham -inoculated and lactating mammary glands (Fig. 5A -2a- compared to -11- and -12-). At 12 h post-IMI with *E. coli* mammary epithelial cells started to protrude from the mammary epithelial layer (Fig. 5A -1b-) resulting in “empty spots” (Fig. 5A: -2b-), and many large cells drifting (Fig. 5A: -2b-) next to the polymorphonuclear cells in the alveolar lumen at 24 h post-IMI (Fig. 5A: -2a- and -3c-). This major histological finding at 12 h post-IMI with *E. coli* resulted in even more cell budding (Fig. 5A -5b-), empty spots (Fig. 5A -5c-), and discarded cells (Fig. 5A: -6b- and -5a-) at 24 h post-IMI. Remarkably, this cell budding phenomenon was far less pronounced in the *S. aureus* infected glands and again clearly delayed as it could only be detected at 24 h post-IMI (Fig. 5A: -10b-).

## Discussion

Infectious mastitis is a complex bacteria-inflicted inflammatory disease that often affects dairy cows. As its traditional antibiotic treatment elicits public controversy and involves human health issues, an increased interest in novel superior therapeutic alternatives has emerged. Therapies that enhance the natural host defense systems by targeting specific pathogens and that limit antibiotic resistance would be very well received in this specific field. Therefore, the detailed molecular description of key signaling modules activated during different types of mammary gland infections is needed but lacking to date. In the current study, an acute murine mastitis model was used to compare the hosts' inflammatory response against the scientifically best documented Gram-negative and Gram-positive bovine mastitis pathogens i.e. *E. coli* strain P4:032 and *S. aureus* strain Newbould 305. Both pathogens multiplied rapidly in the murine mammary gland while triggering the release of pathogen-dependent as well as pathogen-independent immune responses. The different mammary multi-protein patterns each delineated a pathogen-specific infection and occurred concomitantly with discriminatory NF-kappaB activation. More specifically, our data emphasized the importance of evaluating proIL-1beta maturation by complementary immuno-assays as well as IL-1beta KO and caspase-1 KO mice to gain insight in the IL-1beta activity in the mammary gland. While quantitative multiplex analysis showed only the high induction of the classical IL-1beta activity (p17) post-IMI with *S. aureus* compared to *E. coli,* Western blot analysis revealed a differential fragmentation pattern of mammary pro-IL- between both pathogens. This twice involved a non-classical pathway. Infection with *S. aureus* resulted in a later IL-1beta cleavage pattern and was associated with a markedly delayed influx of neutrophils compared to infection with *E. coli*. Furthermore, the mammary epithelium was already damaged at 12 h post-IMI with *E. coli* as characterized by cell budding and empty spots in the architecture, features which were observed later and less prominently post-IMI with *S. aureus*. These data are indicative for a protective cell death mechanism, especially in response to a Gram-negative infection, wherein mammary epithelial cells are massively shed to restrict bacterial growth as well as neutrophil cytotoxicity [26].

Corroborating previous reports generated with either single protein detection analyses (i.e. ELISA) or multi-expression studies (i.e. microarray) of murine mammary glands [27], udder tissue [28] or milk samples [29], our initial quantitative mouse multiplex protein analysis approach demonstrated that an equal count of mammary CFU of *E. coli* and *S. aureus* each induced specific local cytokine profiles. The *E. coli* mediated immune response was characterized by a fast and substantial synthesis of active TNF-alpha, IL-1alpha and MCP-1. These three cytokines were also detected post-IMI with *S. aureus* although only to a relatively minor extent. Intramammary infection with *E. coli* or *S. aureus* was found to equally enhance IL-6 secretion, which can thus be categorized as a Gram-independent immune response. This latter observation corroborated gene expression studies with bovine mammary epithelial cells measuring comparable levels of IL-6 after exposure to both pathogens [21].

In contrast to TNF-alpha, IL-1alpha, MCP-1 and IL-6, the active IL-1beta concentrations at 24 h post-IMI were significantly higher in mammary glands exposed to *S. aureus* compared to *E. coli*. Apparently low levels of IL-1beta were induced at this later time point post-IMI with *E. coli*, nevertheless this pro-inflammatory cytokine was still significantly increased at both time points when compared to sham-inoculated glands and attained its highest level already at 12 h post-IMI. Although this quantitative analysis allowed us to demonstrated different active IL-1beta levels in the mammary gland post-IMI with both pathogens, the proteolytic cleavage of proIL-1beta required for this key proinflammatory mediator to achieve its biological activity remained to be demonstrated as previously stated [30]. Therefore, the presence of IL-1beta fragments was at first verified upon Gram-negative versus the Gram-positive infection by Western blot analysis of the wT mice-derived mammary glands. As suggested by our multiplex data, the classicalp17 IL-1beta cleavage fragment was already detected at 12 h post-IMI with *E. coli* albeit transiently as less p17 was detected at 24 h post-IMI. In contrast, the intensity of the p17 band was much higher at 24 h post-IMI than at 12 h post-IMI with *S. aureus*, indicating slower kinetics for this pathogen. Furthermore, Western blot analysis revealed several additional cleavage fragments besides the classical p17 fragment characterizing the markedly different IL-1beta fragmentation patterns upon infection with both pathogens. More specifically, *E. coli* promoted the formation of a fragment at 25 kDa and two fragments at 15 kDa, while *S. aureus* showed a unique band between 17 kDa and 20 kDa. Both bovine mastitis pathogens induced common bands at 17 kDa, 20 kDa and at 30 kDa. In a subsequent experiment, the comparative analysis of mammary glands from IL-1beta KO mice-compared to wT mice unequivocally confirmed that all these fragments did indeed derive from IL-1beta.

Maturation of pro-IL-1beta is typically controlled by caspase-1 in large multi-molecular complexes called inflammasomes which sense (vita-)MAMPs from live bacteria [22]. To determine whether mammary caspase-1 is also involved in proIL-1beta activation as recently questioned by our group [31], the cleavage of procaspase-1 was investigated. Procaspase-1 fragmentation was clearly visible at 12 h and at 24 h post-IMI with *E. coli*, but unexpectedly far less present at both time points post-IMI with *S. aureus*. This important finding provided a first indication that IL-1beta maturation can also occur independently of caspase-1 in the murine mastitic mammary gland. This caspase-1 independency was confirmed in a subsequent experiment performed to further investigate this process through infection of caspase-1 KO mice. These innovative data strongly supported the hypothesis of a non-conventional maturation of proIL-1beta into active p17 by other proteases than the conventional pro-inflammatory caspase-1, a hypothesis which also was recently postulated by our group [17]. More specifically, the detected protein bands in the IL-1beta fragmentation patterns suggest the likely involvement of secreted neutrophil serine proteases [30]. Indeed, neutrophil elastase and proteinase-3 are known to cleave proIL-1beta (i.e. 31kDa) into fragments of 20 kDa [32] and of 17.5 kDa [30] with different biological activities [30]. Cathepsin [33], another major neutrophil-associated protease, cleaves this immature protein proform directly into two identical fragments of 17.5 kDa [33]. These reported fragments match with the low molecular weight bands retrieved around 20 kDa and p17 for both bovine mastitis pathogens in the current study. The alternative cleavage of pro-IL1beta was detected for *E. coli* at 12 h post-IMI while only at 24 h post-IMI with *S. aureus*. This nicely correlated both with the presence of neutrophils and with local NF-kappa B activation at each of these time points in the mammary gland. The concentrations of two mouse IL-8 like chemokines i.e. KC and MIP-2 were additionally measured in the mammary glands at the relevant time points for each pathogen. As expected, a massive influx of neutrophils was microscopically observed already at 12 h for *E. coli*, while only at 24 h for *S. aureus*. The local chemoattractant concentrations strongly supported this delayed influx as again a milder and slower innate immune response in the mammary gland was seen post-IMI with a Gram-positive compared to a Gram-negative bovine mastitis pathogen.

In contrast to the fragments seen post-IMI with *S. aureus*, *E. coli* infection induced less classical p17 as well as additional non-classical fragment formation upon proIL-1beta cleavage. We here suggest that these fragments can result from matrix metalloproteinases (MMPs) which are secreted by the either mammary epithelial cells [34] or neutrophils [35]. Interestingly, these extracellular enzymes are able to cleave proIL-1beta into either active, or less active, or even non-active IL-1beta fragments [36]. For example, MMP-9 can cleave proIL-1beta into an inactive 26 kDa fragment besides the classical active p17 fragment, while MMP-3 produces inactive 28 kDa as well as less active 14 kDa peptides. In addition, other papers - albeit not in a mammary gland context - describe that MMP-2 can cleave proIL-1beta into both a very low activity 16 kDa and an inactive 10 kDa fragment [37], [38]. At least some of these reported fragments should correspond to fragments from the complex pattern of low-molecular weight bands found in the current study post-IMI with *E. coli* (ranging from 25 kDa to 10 kDa). Importantly however, they do not correspond with the molecular weight of those band found in the current study post-IMI with *S. aureus*. The suggestion that MMPs are induced during the hosts' innate immune response against *E. coli* to inactivate IL-1beta is strengthened by our histological findings [39]. Indeed, the epithelium post-IMI with *E. coli* was clearly damaged as seen on mammary gland sections, a deterioration which again was only mildly present post-IMI with *S. aureus*. Of relevance, a high NF-kappa B activity during mammary gland infection increases caspase-3 mediated cell budding and shedding of epithelial cells [40]. This form of accelerated involution is likely associated with MMPs. Finally, there was also one additional band with a MW between 17.5 kDa and 20 kDa that was selectively present post-IMI with *S. aureus* and not post-IMI with *E. coli*. It is here hypothesized that the latter cleavage fragment might be the product of pathogen-associated proteases (i.e. auto-cleavage) as previously described [41].

Above mentioned arguments implicate the involvement of epithelial (MMPs) and neutrophilic (serine proteases) proteases in the maturation of proIL-1beta. Surprisingly, in both these mammary cell types we could also detect NF-kappaB activity upon immunohistochemical evaluation. Furthermore, it should be highlighted that till date, the precise origin of the mammary proIL-1beta protein remains vague. Nevertheless, from the current study it is clear that the responsible transcription factor inducing the IL-1beta proform is certainly active prior to 12 h post-IMI with both pathogens. However, its maturation occurs only shortly before 12 h post-IMI with *E. coli*, while for *S. aureus* this process occurs about 12 h later i.e. around 24 h. Furthermore, our immunohistochemical data unequivocally demonstrated that for both bacteria the main subunit p65 of the transcription factor NF-kappaB is translocated to the nucleus of the murine mammary epithelial cells. The latter translocation is an essential step for activation of this key inflammatory transcription factor. To evaluate the level of NF-kappaB activation, both pathogens were compared with *in vivo* imaging in the reporter model previously established by our group for *E. coli* [16]. As described for *E. coli* in this latter paper, a fast and strong NF-kappaB activation was again observed in the current study. However, in marked contrast, an on average 3 times lower NF-kappaB activity was detected in the mammary gland for *S. aureus*. Remarkably, the transient enhancement of this NF-kappaB activity already peaked for both pathogens at 6 h post-IMI. This observation highlights that next to the production of TNF-alpha, IL-1alpha and MCP-1, the production of proIL-1beta can be NF-kappa-induced but its activation is likely not. Of relevance in this context, there is still the possibility of other transcription factors which have been reported in udder infections such as activator protein-1 (AP-1) [42], or peroxisome proliferator-activated receptors (PPARs) [43], or regulatory factors such as CAAT box enhancer binding proteins (C/EBPs) [44].

Regardless the underlying transcription mechanism, our data overall imply that proIL-1beta is both highly and differentially processed within 12 h to 24 h post-IMI with both mastitis pathogens, which suggests its tight regulation. The key function of IL-1beta is highlighted by its ability to control the bacterial growth of Gram-negative and to a lesser extend also Gram-positive bacteria. Moreover, the revealed maturation process is certainly also non-classical as it occurs independently from the classical caspase-1 pathway. As a differential IL-1beta fragmentation pattern was observed after Gram-negative compared to Gram-positive mastitis, the cytokine protein profile data from the current study support the hypothesis that *E. coli* and *S. aureus* overall induce a markedly different IL-1beta activity. Our data also corroborate previous findings in KO mice demonstrating that neutrophil IL-1R signaling in mammary gland inflammation mediates neutrophil influx from the interstitium and capillaries into the lumen of the alveoli [13]. Additionally these innate immune cells restrict *E. coli* invasion into the mammary epithelial cells, which is only a characteristic of *S. aureus* infections in wild type mice [9]. We hypothesize that proIL-1beta is locally secreted by the mammary epithelial cells and that multiple fragments likely result from the molding of bacterial proteases and/or NF-kappaB-induced non-caspase proteases secreted from neutrophils (i.e. serine proteases) and mammary epithelial cells (i.e. MMPs). Following infection with both pathogens, the IL-1beta proform is likely present in the interstitium where it is cleaved by serine proteases produced by NF-kappaB activity in neutrophils, which are already sensitized by the preceding release of other pro-inflammatory cytokines following epithelial NF-kappaB signaling. Upon *E. coli* infection in mice, the host rapidly induces an efficient protective program that involves mammary NF-kappaB transcription to enhance TNF-alpha (*E. coli* mediated immune response). Interestingly, TNF-alpha is a well-known inducer of MMPs, which subsequently accelerates mammary gland involution [45], [46]. Subsequently, proIL-1beta becomes inactivated by additional cleavage into fragments abolishing an additional neutrophil influx into the lumen but increasing the number of shedded epithelial cells in order to restrict bacterial growth as well as neutrophil cytotoxicity [26]. This explains the lower IL-1beta levels at 24 h post-IMI versus 12 h post-IMI with *E. coli*. In marked contrast, NF-kappaB is also rapidly but far less activated after *S. aureus* and thus a delayed influx of neutrophils occurs. Moreover, significantly less TNF-alpha is released and thus MMPs will not cause their typical IL-1beta cleavage patterns. In contrast to the early neutrophil-mediated *E. coli* mediated immune response, our data are indicative for the activity of *S. aureus-*derived proteases that alternatively cleave proIL-1beta enabling the typical evasion of these Gram-positive pathogens from the alveolar lumen.

These innovative insights obtained in mice revealed at first a sentinel function for IL-1beta during the early innate inflammatory response to mammary gland infections. Additionally, they highlight the limitations of single analysis methods. To acknowledge the proposed novel molecular mechanism of proIL-1beta in the early innate immune signaling during mastitis, additional studies are mandatory. Attractive options to extend our current findings are either the use of acute models with other relevant mastitic germs [47], or of more complex chronic mouse mastitis models [48] and last but not least of different mammalian target species, especially dairy animals.

## Supporting Information

Figure S1 **Secondary host response post-IMI with** ***E. coli*** **or** ***S. aureus*** **.** IL-6 and KC blood values post-IMI with *E. coli* and *S. aureus* compared to the sham-inoculated (PBS) and lactating (0 h) negative controls. (A) Hepatic IL-1alpha, IL-6 and KC values post-IMI with *E. coli* or with *S. aureus* compared to sham-inoculated (PBS) negative controls. (B) Changes in core body temperature post-IMI with *E. coli* or with *S. aureus*. (C) Letters of homogeneous subsets were marked if the difference between treatments were statistically significant (P<0.05). DL = detection limit.
(TIF)

Figure S2 **Hepatic NF-kappaB activity in transgenic reporter mice post-IMI with** ***E. coli*** **or** ***S. aureus*** **.** (A) *In vivo* imaging follow-up of NF-kappaB activity in the liver (ventral view) after an IMI with *E. coli* or *S. aureus*. The data represents the flux density (total flux radiance per selected body area (in p*(s*m^2^))). (B) Representative photograph of lactating mice (most left) compared to sham-inoculated (PBS, 2nd from left) mice at 24 h post-IMI with *S. aureus* (3rd from left), or *E. coli* (most right). The intensity of luminescence was scaled based on the radiance (in p*(s*m^2^)). (C) Livers of NF-kappaB reporter mice were excised at 12 h and at 24 h post-IMI, and their luminescence was measured *ex vivo.* The graph represents the total flux radiance per area (in p*(s*m^2^)) as a measure for the NF-kappaB activity. (D) Representative photographical view of excised livers post-IMI with *E. coli* (top), *S. aureus* (middle) or PBS (bottom). Letters of homogeneous subsets were marked if the difference between treatments were statistically significant (P<0.05).
(TIF)

Figure S3 **Differential nuclear translocation of mammary NF-kappaB p50 post-IMI with** ***E. coli*** **vs** ***S. aureus*** **.** Similar as for the p65 subunit of NF-kappaB, nuclear translocation of the mammary NF-kappaB p50 subunit was detected post-IMI with both pathogens. Interestingly, the translocation of p50 for *S. aureus* was only observed at 24 h post-IMI but not 12 h post-IMI, while at both time points p50 was detected in the nucleus post-IMI with *E. coli*.
(TIF)

Text S1 **Addendum text describing the extra-mammary changes in mice during an intramammary infection with** ***S. aureus*** **and** ***E. coli.***
(DOCX)
