# Supplementary figures and images for: Non-Classical ProIL-1beta Activation during Mammary Gland Infection Is Pathogen-Dependent but Caspase-1 Independent
Source: PLoS One. 2014 Aug 27;9(8):e105680. doi: 10.1371/journal.pone.0105680 (PMC4146512; doi:10.1371/journal.pone.0105680)

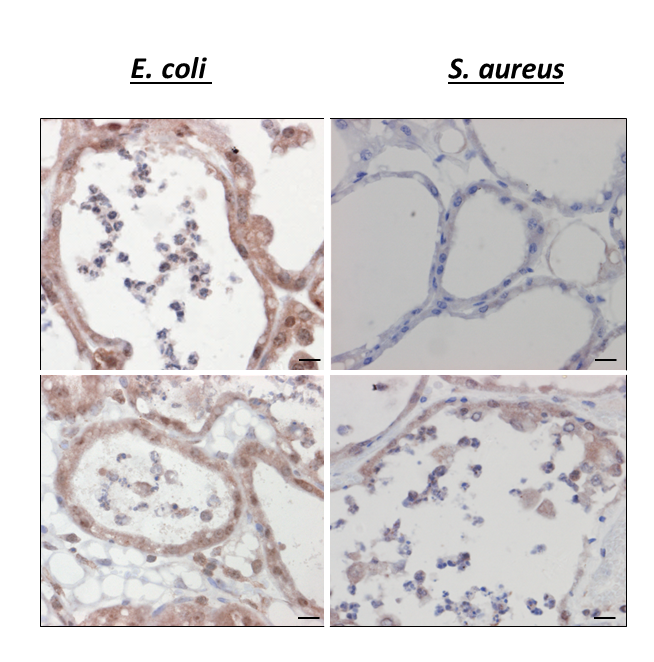

Supplement: Figure S3 — Differential nuclear translocation of mammary NF-kappaB p50 post-IMI with E. coli vs S. aureus . Similar as for the p65 subunit of NF-kappaB, nuclear translocation of the mammary NF-kappaB p50 subunit was detected post-IMI with both pathogens. Interestingly, the translocation of p50 for S. aureus was only observed at 24 h post-IMI but not 12 h post-IMI, while at both time points p50 was detected in the nucleus post-IMI with E. coli. (TIF) [file pone.0105680.s003.tif]
