# Supplementary material for: Non-Classical ProIL-1beta Activation during Mammary Gland Infection Is Pathogen-Dependent but Caspase-1 Independent
Source: PLoS One. 2014 Aug 27;9(8):e105680. doi: 10.1371/journal.pone.0105680 (PMC4146512; doi:10.1371/journal.pone.0105680)
Supplement: Text S1 — Addendum text describing the extra-mammary changes in mice during an intramammary infection with S. aureus and E. coli. (DOCX) [file pone.0105680.s004.docx]

**SUPPORTING INFORMATION**

Differences secondary to the local immune response in mice post-IMI with *E. coli* versus post-IMI with *S. aureus* could be detected based on serum and hepatic cytokines, hepatic NFkappaB-activity and body temperature.

The levels of IL-6 and KC in blood were marked by a response post-IMI with *S. aureus* or post-IMI with *E. coli,* respectively (Fig. S1A). In contrast to the mammary response, IL-6 was significantly higher post-IMI with *S. aureus* compared to sham-inoculated (PBS) glands. This observation could be linked to the susceptibility of mice to the virulence of *S. aureus* because temperature regulation is associated with systemic IL-6 levels [[1](#_ENREF_1),[2](#_ENREF_2),[3](#_ENREF_3)] (see further). Therefore, the higher increase in systemic IL-6 levels at 24h post-IMI is associated with the hypothermia post-IMI with *S. aureus* (Fig. S1C)*.* The KC levels were significantly higher post-IMI with *E. coli* compared to PBS. It is known that a chemotactic gradient of KC guides blood neutrophils into the mammary alveolar lumen [[4](#_ENREF_4)]. As such, this observation is in line with the earlier detected neutrophil influx post-IMI with *E. coli* compared to *S. aureus.* Furthermore, levels of TNF-alpha, IL-1alpha, MCP-1 and IL-1beta in the blood were not elevated compared to the sham-inoculated mice.

As shown in Fig. S1B and Fig.S2, elevated cytokines and NF-kappaB transcription were also detected in the liver at 24h post-IMI both pathogens compared to sham-inoculated (PBS) mice. Similarly as in the blood, hepatic IL-6 and KC were also elevated post-IMI with *S. aureus* and post-IMI with *E. coli* compared to the sham-inoculated mice. As the liver controls the homeostasis, hepatic production of cytokines [[5](#_ENREF_5)] and acute phase proteins [[6](#_ENREF_6)] are likely to control the systemic inflammatory responses [[7](#_ENREF_7)]. As already suggested by our group post-IMI with *E. coli* [[8](#_ENREF_8)], both *E. coli* and *S. aureus* induced a high NF-kappaB activity in the liver.

The mice were significantly influenced by the *S. aureus* pathogenicity in contrast to *E. coli* or sham-inoculated (PBS) mice as reflected in the drop in body temperature (Fig. S1C). This observation is in line with previous reports. Corroborating characteristics of nearly all *S. aureus* strains, this subspecies is characterized by hemolysin and coagulase activity [[9](#_ENREF_9),[10](#_ENREF_10),[11](#_ENREF_11),[12](#_ENREF_12)]. Both these exotoxins are associated with high virulence in the mouse mastitis model [[13](#_ENREF_13)]. Mice are in particular sensitive to the alpha-hemolysin/alpha-toxin (*hla*), especially when *S. aureus* bacteria are introduced in the mammary gland at a high inoculum dose. This was elegantly demonstrated by the fact that depletion of the toxin in the *S. aureus* strain -when inoculated at the same high dose- completely reversed the *S. aureus* lethality during mastitis in mice [[14](#_ENREF_14)].

**REFERENCES**

1. Chai Z, Gatti S, Toniatti C, Poli V, Bartfai T (1996) Interleukin (IL)-6 gene expression in the central nervous system is necessary for fever response to lipopolysaccharide or IL-1 beta: a study on IL-6-deficient mice. J Exp Med 183: 311-316.

2. Stewart CR, Landseadel JP, Gurka MJ, Fairchild KD (2010) Hypothermia increases interleukin-6 and interleukin-10 in juvenile endotoxemic mice. Pediatr Crit Care Med 11: 109-116.

3. Shalaby MR, Waage A, Aarden L, Espevik T (1989) Endotoxin, tumor necrosis factor-alpha and interleukin 1 induce interleukin 6 production in vivo. Clin Immunol Immunopathol 53: 488-498.

4. Li Jeon N, Baskaran H, Dertinger SK, Whitesides GM, Van de Water L, et al. (2002) Neutrophil chemotaxis in linear and complex gradients of interleukin-8 formed in a microfabricated device. Nat Biotechnol 20: 826-830.

5. van Meijl LE, Popeijus HE, Mensink RP (2010) Amino acids stimulate Akt phosphorylation, and reduce IL-8 production and NF-kappaB activity in HepG2 liver cells. Mol Nutr Food Res 54: 1568-1573.

6. Trautwein C, Boker K, Manns MP (1994) Hepatocyte and Immune-System - Acute-Phase Reaction as a Contribution to Early Defense-Mechanisms. Gut 35: 1163-1166.

7. Tacke F, Luedde T, Trautwein C (2009) Inflammatory pathways in liver homeostasis and liver injury. Clin Rev Allergy Immunol 36: 4-12.

8. Notebaert S, Carlsen H, Janssen D, Vandenabeele P, Blomhoff R, et al. (2008) In vivo imaging of NF-kappaB activity during Escherichia coli-induced mammary gland infection. Cell Microbiol 10: 1249-1258.

9. Bhakdi S, Tranum-Jensen J (1991) Alpha-toxin of Staphylococcus aureus. Microbiol Rev 55: 733-751.

10. Dinges MM, Orwin PM, Schlievert PM (2000) Exotoxins of Staphylococcus aureus. Clinical Microbiology Reviews 13: 16-+.

11. Bouchard D, Peton V, Almeida S, Le Marechal C, Miyoshi A, et al. (2012) Genome sequence of Staphylococcus aureus Newbould 305, a strain associated with mild bovine mastitis. J Bacteriol 194: 6292-6293.

12. Brouillette E, Grondin G, Lefebvre U, Talbot BG, Malouin F (2004) Mouse mastitis model of infection for antimicrobial compound efficacy studies against intracellular and extracellular forms of Staphylococcus aureus. Veterinary Microbiology 101: 253-262.

13. Jonsson P, Lindberg M, Haraldsson I, Wadstrom T (1985) Virulence of Staphylococcus aureus in a mouse mastitis model: studies of alpha hemolysin, coagulase, and protein A as possible virulence determinants with protoplast fusion and gene cloning. Infect Immun 49: 765-769.

14. Bramley AJ, Patel AH, O'Reilly M, Foster R, Foster TJ (1989) Roles of alpha-toxin and beta-toxin in virulence of Staphylococcus aureus for the mouse mammary gland. Infect Immun 57: 2489-2494.
